# Editorial: Nutrition and metabolism in cancer: role in prevention and prognosis

**DOI:** 10.3389/fnut.2025.1631547

**Published:** 2025-06-10

**Authors:** Lucilla Crudele, Antonio Moschetta, Jean-Marc Lobaccaro

**Affiliations:** ^1^Department of Interdisciplinary Medicine, University of Bari Aldo Moro, Bari, Italy; ^2^Institut GReD, Université Clermont Auvergne, Clermont-Ferrand, France

**Keywords:** cholesterol, hepatocellular carcinoma, obesity, adipose tissue, metabolic pathway

The intricate relationship between nutrition and cancer continues to be a critical area of research, influencing patient prognosis, treatment tolerance, and overall quality of life (1). As our understanding deepens, the focus shifts from general dietary guidelines to more personalized nutritional assessments, interventions, and the identification of specific nutritional markers that can predict patient outcomes. This Research Topic compiles a collection of 30 articles that delve into various facets of this complex interplay, offering valuable insights on key areas of progress, ranging from the prognostic value of nutritional and inflammatory indices to the impact of body composition, specific dietary components, and targeted nutritional support strategies across various cancer types. Obesity and fat mass excess have been investigated as negative prognostic factors. Specifically, adipose tissue have been investigated as potential new prognostic markers in patients with locally advanced head and neck cancer by Carrilho et al. Indeed, while the Body Mass Index (BMI) provides a holistic representation of overall weight, it lacks ability to differentiate between muscle and fat proportion. Thus, Zhao et al. demonstrated that evaluating fat distribution is useful for assessing obesity in cancer patients, aiding surgical planning and post-operative care to reduce complications and improve overall survival. Adipose tissue is responsible for derangement in metabolic pathways as well as low-grade chronic inflammation.

The involvement of dysmetabolic conditions in cancer has also been demonstrated by Zhang, Shao et al. showing the prognostic value of another metabolic marker, the triglyceride-glucose index, in non-muscle-invasive bladder cancer. The role of cholesterol in cancer pathogenesis has been extensively demonstrated from a molecular point of view (2). Here, hyperlipidemia, assessed through the triglycerides-glucose index, has been confirmed by Wang, Zhu et al. as a prognostic factor for patients with newly diagnosed epithelial ovarian cancer. Also the non-high-density lipoprotein to high-density lipoprotein ratio (NHHR) was found strongly associated with all-cause mortality in cancer survivors, by Xie et al. who analyzed cancer survivor data from the National Health and Nutrition Examination Survey (NHANES) from 1999 to 2018.

Beyond composite scores, individual biochemical markers and inflammatory indicators are also extensively explored for their prognostic value. Serum albumin is repeatedly identified as a crucial marker. Duan et al. considered age, serum albumin levels, BMI, and activities of daily living (ADL) to propose a new prediction model that demonstrates remarkable accuracy in forecasting malnutrition in elderly hospitalized cancer patients. Lower albumin levels have been linked to frailty measures, trace elements, and inflammation. Inflammatory markers, such as the Neutrophil-to-Lymphocyte Ratio (NLR), Platelet-to-Lymphocyte Ratio (PLR), and Systemic Immune-Inflammation Index (SII), which often combine blood cell counts, are also featured. These indices are suggested to be significant predictive factors for the development of malnutrition in patients with colorectal cancer in the review by Wang, Tang et al. while a preoperative NLR-based prognostic model has been applied by Qi et al. to predict the prognosis of intrahepatic cholangiocarcinoma following radical surgery, categorizing patients into different prognostic groups based on NLR cutoffs. The C-reactive protein to albumin ratio (CAR) is proposed as a potentially good prognostic marker in patients undergoing hepatectomy for hepatocellular carcinoma (HCC), according to a meta-analysis by Wang, Xu et al. An Empirical Dietary Inflammatory Pattern (EDIP) with higher inflammatory properties has been found to be associated with increased cancer risk and cancer-specific mortality by Hosseini et al. Pro-inflammatory foods include processed meat, red meat, refined grains, and high-energy drinks. Conversely, tomato and pizza showed inverse associations with inflammatory markers in some contexts.

However, although obesity may represent a risk factor for cancer development and worse prognosis, on the other hand, malnutrition itself is a significant concern in oncology. Wang, Liu et al. explored the application and validity of the Global Leadership Initiative on Malnutrition (GLIM) (3) criteria in different cancer types and clinical settings, concluding that they appear to require further investigation, particularly in specific populations like East Asian patients with gastric cancer. Conversely, in the context of colorectal cancer patients post-radical surgery, Yan et al. developed and validated a novel prognostic prediction system based on GLIM-defined malnutrition finding that malnutrition was associated with total complications.

A meta-analysis covering studies from 2016 to 2024, primarily from Asia, assessed the Prognostic Nutritional Index (PNI)'s impact on overall survival (OS), recurrence-free survival, and disease-free survival in patients with hepatocellular carcinoma after curative hepatectomy. Intriguingly, Zhang, Li et al. speculate that HCC patients could potentially benefit from preoperative treatments, such as enteral nutrition support or non-steroid anti-inflammatory drugs, to achieve a more favorable PNI value. Furthermore, a higher PNI (suggesting no or mild nutritional risk) was associated with better prognosis compared to severe nutritional risk in prostate cancer patients by Li, Wan et al. and as a predictive factor for malnutrition in colorectal cancer (CRC) patients by Wang, Tang et al. Nutritional monitoring including physical indicators like weight, percentage weight change, BMI, and grip strength, as well as laboratory indices such as serum albumin, prealbumin, electrolytes, and C-reactive protein (CRP) are recommended by Fan et al. in patients receiving radiotherapy for nasopharyngeal carcinoma.

Sarcopenia, characterized by low appendicular lean mass (ALM) and low hand grip strength (HGS), has been associated with an increased risk of HCC in the European population in a Mendelian randomization study by Cao et al. The association between dietary creatine intake and cancer in US adults has been studied by Jiang et al., but limitations include potentially lower creatine intake in cancer patients due to recommendations to reduce red meat consumption and the exclusion of non-animal protein sources.

In this context, improvement of nutritional strategies in oncology is mandatory.

The use of oral nutritional supplements (ONS) to control weight loss in postoperative patients with solid tumors (gastric, colorectal, head and neck, or bladder cancer) has been reviewed by Liu, Wu et al. and specific dietary factors have been examined for their association with cancer risk or prognosis, although therapeutic controversies over the use of antioxidant supplements during cancer treatment are also reviewed by Woldeselassie and Tamene. For instance, a systematic review by Martínez-Domínguez et al. found no association between vitamin C intake (dietary/supplements or circulating levels) and pancreatic cancer risk, suggesting no specific recommendation for high vitamin C intake for this purpose is needed while a more recent meta-analysis by Ni et al. on the association between dietary antioxidant vitamins (A, C, E) and glioma risk found that higher intake of vitamin C significantly reduced glioma risk, while no significant associations were observed for vitamin A or E.

In conclusion, this topic offers a multifaceted view of the relationship between nutrition and cancer, underscoring the predictive value of various nutritional and body composition markers, examining the effectiveness of different forms of nutritional support, and analyzing the impact of dietary patterns and specific nutrients on cancer risk and prognosis. They also highlight the methodological challenges in nutritional oncology research and the need for further high-quality studies to strengthen the evidence base.
